# A deep learning masked segmentation alternative to manual segmentation in biparametric MRI prostate cancer radiomics

**DOI:** 10.1007/s00330-022-08712-8

**Published:** 2022-04-14

**Authors:** Jeroen Bleker, Thomas C. Kwee, Dennis Rouw, Christian Roest, Jaap Borstlap, Igle Jan de Jong, Rudi A. J. O. Dierckx, Henkjan Huisman, Derya Yakar

**Affiliations:** 1 Medical Imaging Center, Departments of Radiology, Nuclear Medicine and Molecular Imaging, University Medical Center Groningen, University of Groningen, Meditech Building, Room 305, Hanzeplein 1, 9700 RB Groningen, The Netherlands; 2 Department of Radiology, Martini Hospital Groningen, Van Swietenplein 1, 9728 NT Groningen, The Netherlands; 3 Department of Radiology, Treant Zorggroep, Dr. G.H. Amshoffweg 1, 7909 AA Hoogeveen, The Netherlands; 4 Department of Urology, University Medical Center Groningen, University of Groningen, Hanzeplein 1, 9700 RB Groningen, The Netherlands; 5 Department of Radiology and Nuclear Medicine, Radboud University Medical Center, Geert Grooteplein Zuid 10, 6525 GA Nijmegen, The Netherlands

**Keywords:** Biomarkers, Deep learning, Prostatic neoplasms, Multi-center study, Data curation

## Abstract

**Objectives:**

To determine the value of a deep learning masked (DLM) auto-fixed volume of interest (VOI) segmentation method as an alternative to manual segmentation for radiomics-based diagnosis of clinically significant (CS) prostate cancer (PCa) on biparametric magnetic resonance imaging (bpMRI).

**Materials and methods:**

This study included a retrospective multi-center dataset of 524 PCa lesions (of which 204 are CS PCa) on bpMRI. All lesions were both semi-automatically segmented with a DLM auto-fixed VOI method (averaging < 10 s per lesion) and manually segmented by an expert uroradiologist (averaging 5 min per lesion). The DLM auto-fixed VOI method uses a spherical VOI (with its center at the location of the lowest apparent diffusion coefficient of the prostate lesion as indicated with a single mouse click) from which non-prostate voxels are removed using a deep learning–based prostate segmentation algorithm. Thirteen different DLM auto-fixed VOI diameters (ranging from 6 to 30 mm) were explored. Extracted radiomics data were split into training and test sets (4:1 ratio). Performance was assessed with receiver operating characteristic (ROC) analysis.

**Results:**

In the test set, the area under the ROC curve (AUCs) of the DLM auto-fixed VOI method with a VOI diameter of 18 mm (0.76 [95% CI: 0.66–0.85]) was significantly higher (*p* = 0.0198) than that of the manual segmentation method (0.62 [95% CI: 0.52–0.73]).

**Conclusions:**

A DLM auto-fixed VOI segmentation can provide a potentially more accurate radiomics diagnosis of CS PCa than expert manual segmentation while also reducing expert time investment by more than 97%.

**Key Points:**

• *Compared to traditional expert-based segmentation, a deep learning mask (DLM) auto-fixed VOI placement is more accurate at detecting CS PCa.*

• *Compared to traditional expert-based segmentation, a DLM auto-fixed VOI placement is faster and can result in a 97% time reduction.*

• *Applying deep learning to an auto-fixed VOI radiomics approach can be valuable.*

**Supplementary Information:**

The online version contains supplementary material available at 10.1007/s00330-022-08712-8.

## Introduction

Prostate cancer (PCa) remains the most common cancer among western males [1]. Multiparametric magnetic resonance imaging (mpMRI) can be useful for the detection of clinically significant (CS) PCa and its discrimination from non-significant entities [2]. However, qualitative mpMRI interpretation requires extensive experience [3] and may miss some significant cancers [2].

Recent studies have shown promising results of radiomics models for CS PCa diagnosis [4]. The far majority of the studies on this topic used labor-intensive manual segmentation to extract radiomics features [5]. Moreover, most clinical studies on radiomics used relatively small annotated single-center datasets (*n* < 300) [6], which might be related to the time-consuming nature of manual segmentations. Prostate cancer imaging studies can have a diverse approach to segmentation. Some do not require any manual lesion segmentation and instead use or segment the lesion or different areas (e.g., entire image, whole gland or prostate zones) with the use of fully automated deep learning algorithms [7]. Other segmentation options include semi-automated (combination of manual and automated) approaches or manual expert-based segmentation [8]. Radiomics mostly involves manual segmentation of a volume of interest (VOI) [5] with the extracted features giving specific information about the structure inside the VOI, e.g., the PCa lesion. This allows for the quantification of tumor phenotypes using imaging biomarkers which might facilitate the construction of explainable AI models. However, manual segmentation is a tedious and time-consuming task so a new segmentation method is required to more rapidly extract radiomics features. This will facilitate the execution of larger (multi-center) studies to improve the diagnostic performance and generalizability of radiomics models [9–11]. This might eventually increase the willingness of radiologists and clinicians to implement an mpMRI–based radiomics analysis for CS PCA in daily workflows.

**Fig. 1:**
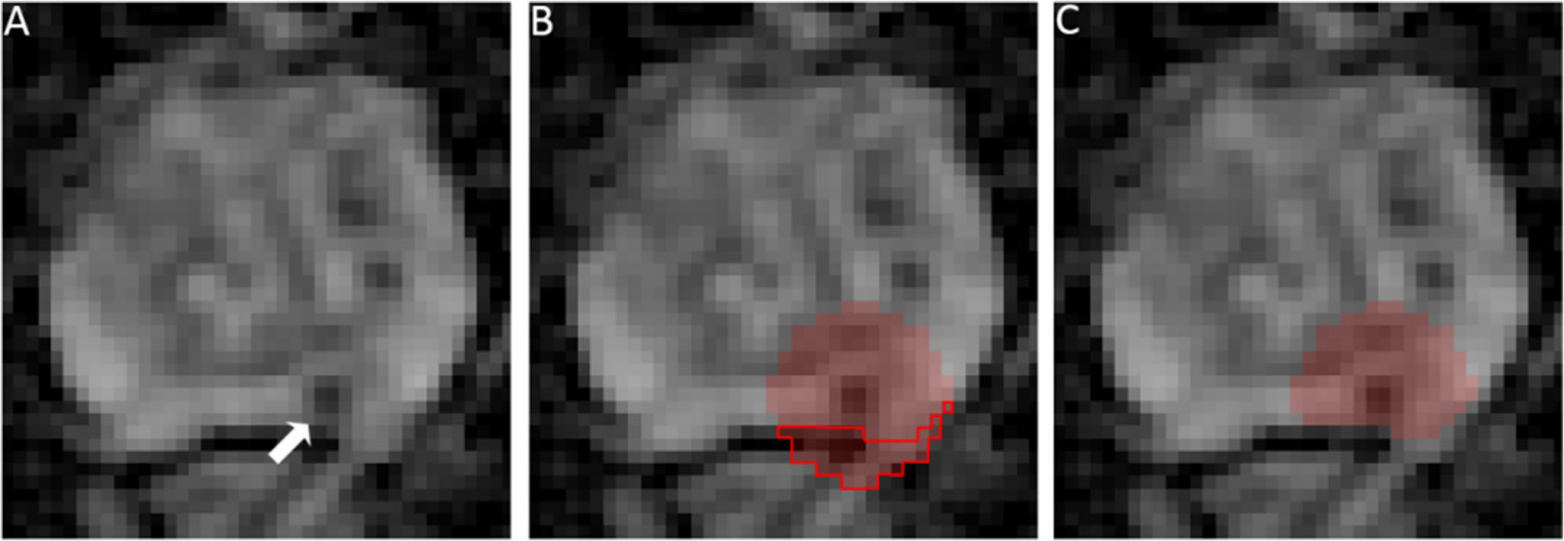
**A** Apparent diffusion coefficient map image (3× zoom factor) in a 76-year-old man with a suspicious lesion in the peripheral zone indicated by the arrow (PI-RADS 4) that proved to be an ISUP grade 2 PCa (based on MRI-TRUS fusion). **B** 18-mm auto-fixed VOI placed around the voxel with the lowest ADC value; due to the location of the lesion, a large number of voxels outside the prostate are included (red outline). **C** Result of auto-fixed VOI combined with deep learning–based segmentation to remove unwanted voxels outside the prostate

In a previous study [12], we showed that radiomics features extracted from an mpMRI dataset based on a spherical volume of interest (VOI) placed around the lesion voxel with the lowest apparent diffusion coefficient (ADC) (“auto-fixed VOI”) can be a valuable addition to visual assessment in diagnosing CS PCa [12]. The auto-fixed VOI can substantially reduce an expert’s post-processing time because it only requires the selection of the voxel with the visually lowest ADC value, which can be done in a matter of seconds. Also, the influence of the radiologist can be reduced if the voxel with the lowest ADC value is detected quantitatively. With semi-automated detection, the matrices for texture features are always centered around the area with the lowest ADC, consequently normalizing the VOI delineation and feature calculation. However, the semi-automated sphere placement in the area around the reference point that is indicated by the expert will occasionally include voxels outside the prostate, which introduces unwanted information in the radiomics model (Fig. 1A, B). We propose combining the auto-fixed VOI method with a deep learning–based segmentation that removes all unwanted voxels from the VOI that are located outside the prostate (Fig. 1C). Since the deep learning masked (DLM) auto-fixed VOI is not a precise delineation of the lesion like a manual segmentation, the auto-fixed VOI will contain areas with the edge of the lesion and healthy prostate tissue (i.e., the transition of lesion tissue to healthy prostate tissue). The edge and healthy tissue information could potentially be helpful for radiomics texture and intensity–based features. Gradient and sharpness of lesion edges might contain interesting information while the addition of some healthy tissue allows for minor intensity correction. We hypothesize that the DLM auto-fixed VOI is faster and non-inferior to a completely manually segmented VOI in a bpMRI radiomics model for the diagnosis of CS PCa.

Therefore, the purpose of this study was to determine the value of a DLM auto-fixed VOI segmentation method as an alternative to manual segmentation for radiomics-based diagnosis of CS PCa on bpMRI.

## Materials and methods

### Patient data

The patient data for this study (*n* = 930) consisted of a multi-center dataset from 9 different medical centers (2 tertiary care academic institutions, 7 non-academic institutions). All patients from the 7 non-academic institutions (along with their MRI, pathological, and all other clinical data) were referred to the 2 tertiary care academic centers for image-guided biopsy as part of clinical care. The institutional review board of the 2 tertiary care academic institutions approved this study and waived the need for informed consent. Detailed description of settings, scanners, and lesion grading for the 930 patients in the multi-center dataset can be found in Electronic supplementary material 1.

### Patient characteristics and lesion selection

**Fig. 2:**
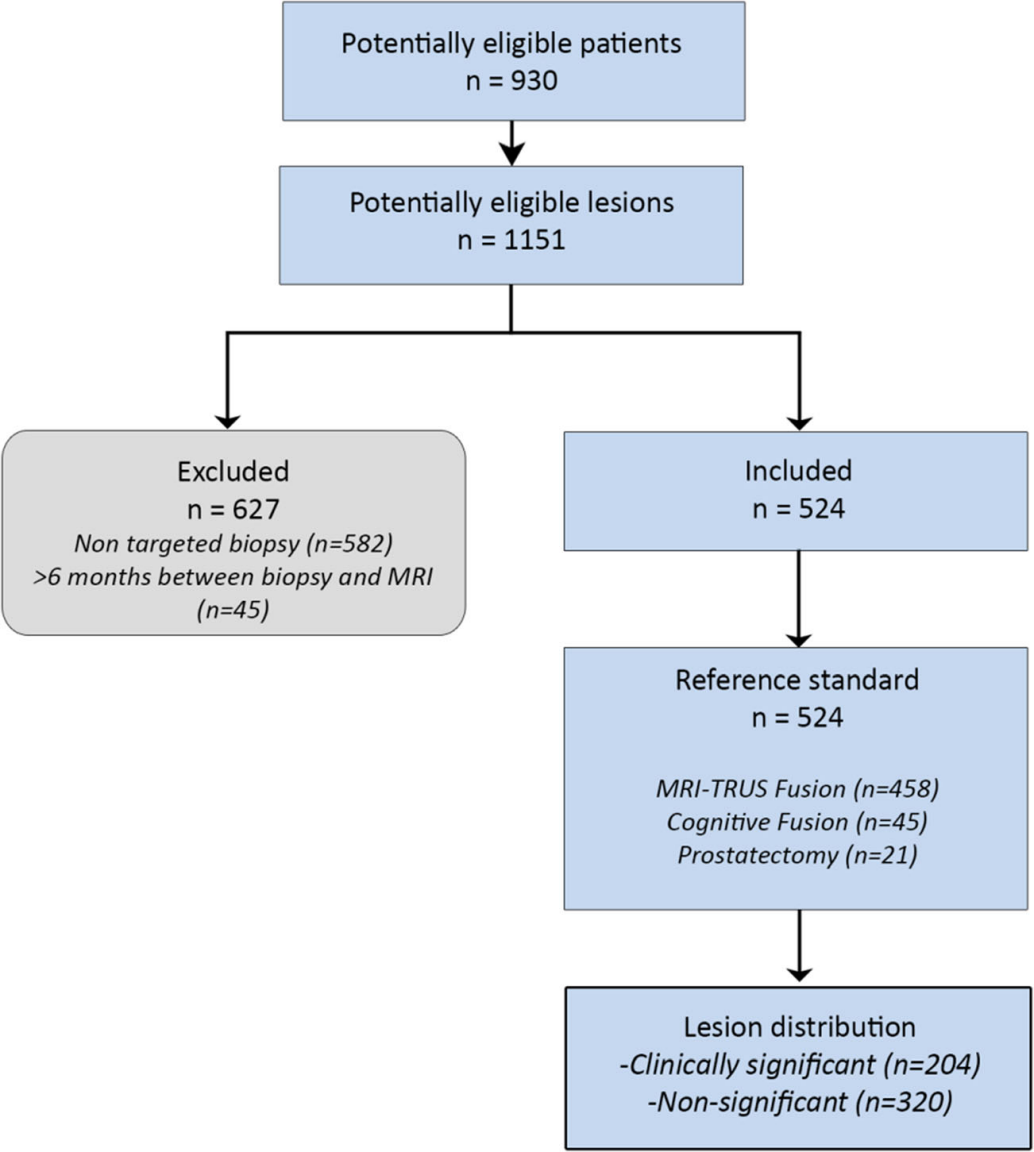
Patient and lesion selection flowchart

Lesions from the 930 patients were included if a corresponding ISUP grade could be established based on available targeted biopsy (TRUS fusion biopsy, cognitive fusion biopsy, in-bore MRI targeted) or prostatectomy specimens performed within a maximum of 6 months after MRI. CS PCa was defined as International Society of Urological Pathology (ISUP) grade ≥ 2. Lesions were excluded if ISUP grading could only be done based on non-targeted TRUS biopsy, and when biopsy was done before MRI. Of the 1151 lesions that were assigned a PI-RADS score, 524 lesions from 427 patients were eventually included (Fig. 2). Patient information and details on the biopsy and grading for the study population can be found in Electronic supplementary material 2.

### DLM auto-fixed VOI: development and optimization

**Fig. 3:**
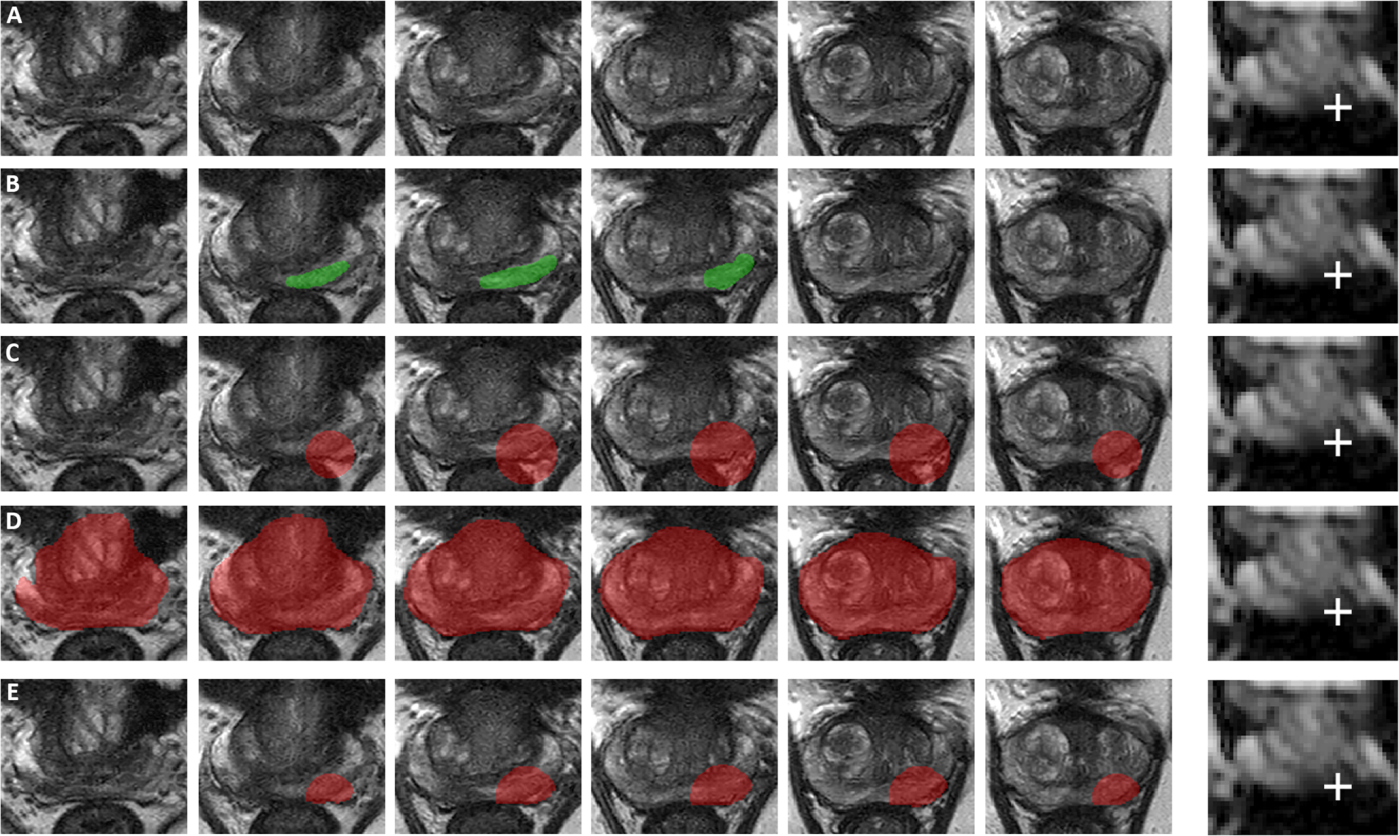
Axial T2-weighted images in a 73-year-old man with a suspicious lesion in the peripheral zone (PI-RADS 4, mostly based on DWI [third slice of ADC map containing the lesion attached for reference with a white cross indicating the single-click voxel with the quantitatively acquired lowest ADC value]) that proved to be an ISUP grade 3 PCa (based on MRI-TRUS fusion, confirmed by prostatectomy). **A** T2-weighted images without any segmentation. **B** Results of slice-by-slice manual lesion segmentation by an expert uroradiologist. **C** Results of 18-mm auto-fixed VOI lesion segmentation without DLM adjustment. **D** Deep learning–based total prostate segmentation. **E** Results of 18-mm auto-fixed VOI lesion segmentation with DLM adjustment

The previously reported auto-fixed VOI method extracts radiomics features from a bpMRI dataset based on a spherical VOI placed around the lesion voxel with the lowest ADC [12]. However, this method suffers from the inclusion of voxels outside the prostate (Fig. 3). To eliminate voxels outside the prostate, a deep learning algorithm that segments the total prostate was added to limit the auto-fixed VOI to the boundaries of the prostate. As such, a DLM auto-fixed VOI method for the extraction of radiomics features was developed. The deep learning algorithm used the 3D U-Net architecture [13] and was trained on the open-source multi-site dataset for prostate MRI segmentation [14] (*n* = 116). An open-source dataset was used due to the need for an accurate and tested total prostate segmentation dataset. The 3D U-Net was trained using Tensorflow/Keras version 2.2.0 on a 32-GB V100 GPU. The U-Net was built up of convolutional blocks containing 2 consecutive 3D convolution operations with a kernel size of 3 × 3 × 3, and contained three downsampling and upsampling operations. Max pooling (3 × 3 × 2) was used to downsample the image in the contracting pathway of the model, while upsampling was performed with nearest neighbor interpolation, also with a kernel size of 3 × 3 × 2. After each lower image scale, the number of computed filters was doubled, starting at 64 in the initial convolutional block. A maximum of 400 epochs was used with early stopping after 40 epochs if the validation loss did not improve. Batch size was set to 1 due to memory restrictions on the GPU. An Adam optimizer [15] was used with an initial learning rate of 1e−4. No weight decay was implemented; however, L2 regularization with a factor of 1e−4 was applied to the kernels of each convolutional layer. The model used T2-weighted images as input which were resampled to a voxel spacing of 0.5 mm with a slice thickness of 3 mm with linear interpolation, and subsequently cropped to a volume of 135 × 135 × 14. *Z*-score normalization was applied to each image, which adjusted each image voxel intensity distribution to a mean of 0 and standard deviation of 1. Visual assessment of the prostate segmentations was successful on 10% random sample to validate the correctness of the segmentation of the total prostate

(Fig. 4).

**Fig. 4:**
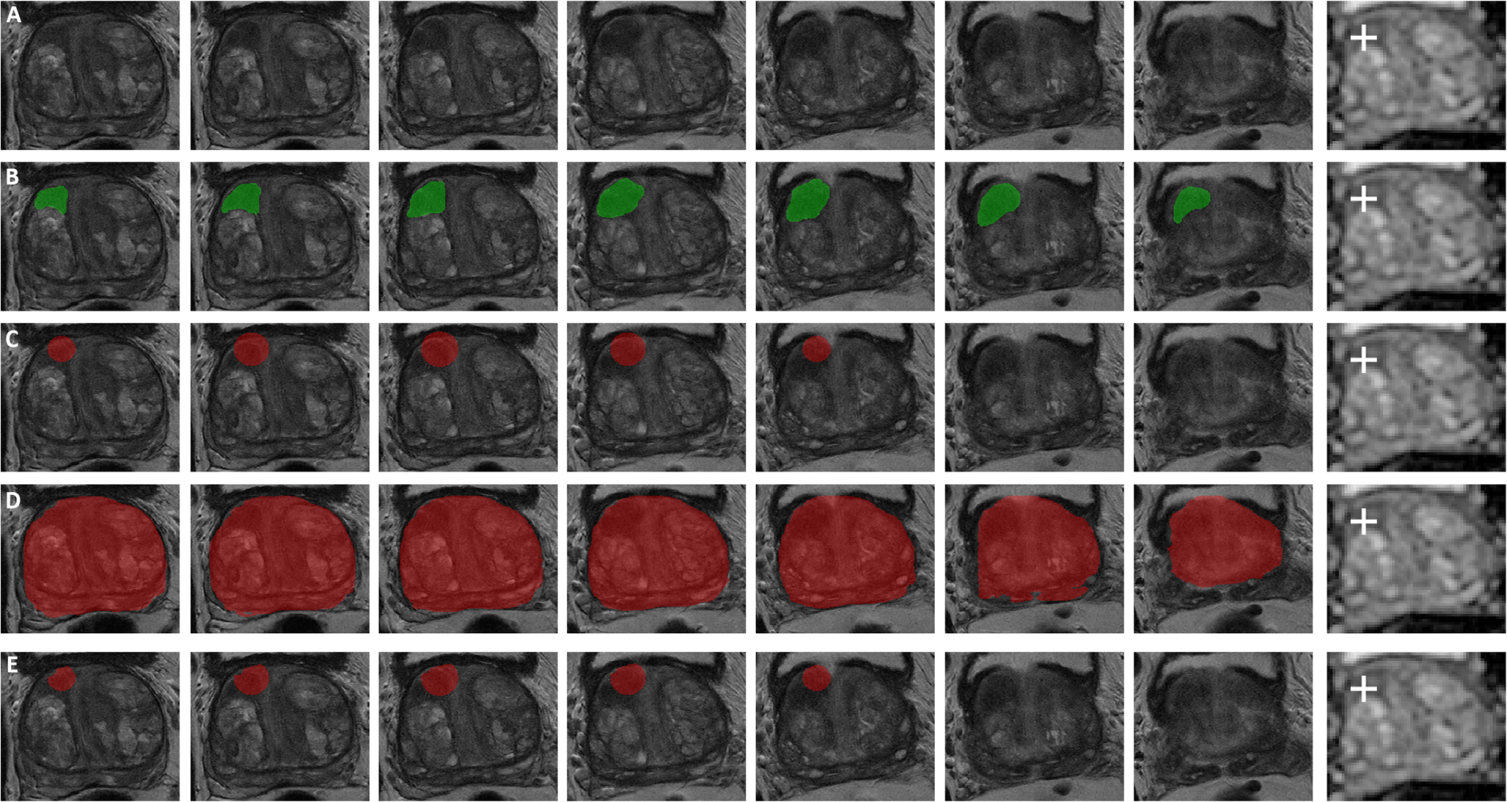
Axial T2-weighted images in a 74-year-old man with a suspicious lesion in the transition zone (PI-RADS 3, mostly based on DWI [third slice of ADC map containing the lesion attached for reference with a white cross indicating the single-click voxel with the quantitatively acquired lowest ADC value]) that proved to be non-significant PCa (based on MRI-TRUS fusion). **A** T2-weighted images without any segmentation. **B** Results of slice-by-slice manual lesion segmentation by an expert uroradiologist. **C** Results of 18-mm auto-fixed VOI lesion segmentation without DLM adjustment. **D** Deep learning–based total prostate segmentation. **E** Results of 18-mm auto-fixed VOI lesion segmentation with DLM adjustment

The DLM auto-fixed VOI method allows the (uro)radiologist to automatically create a customized VOI by a single mouse click on the location that visually has the lowest ADC, a process that generally takes less than 10 s since the radiologist only has to select the visually lowest ADC part of the lesion on a single slice. To ensure a consistent selection of the exact voxel with the lowest ADC (i.e., quantitatively acquiring the voxel with the lowest intensity), automatic hyperbolic VOI centering occurred in the area around the single mouse click. An algorithm checked the 3D neighborhood (56-connectivity) of the mouse click and, if needed, applied automatic repositioning to the voxel with the lowest ADC value without being affected by single voxel outliers. Due to the relative ease of visually selecting the area where the lowest ADC voxel should be located and the following 3D neighborhood check, a quantitative selection of the correct voxel was acquired*.* A sphere is constructed around this voxel and masked using the total prostate segmentation. A segmentation quality control was added to ensure that the masking did not remove the index voxel (lowest ADC finding algorithm) since this voxel is always located inside the lesion and therefore within the prostate. This quality check was never triggered confirming the quality of the prostate segmentation algorithm. Figure 3 shows an example of DLM auto-fixed VOI segmentation. Next, in order to optimize the size of the fixed diameter of the initial spherical VOI, the effect of various diameters on the diagnostic performance of the bpMRI radiomics model was tested. For this purpose, diameters ranging from 6 to 30 mm with 2-mm increments were used. The size of these diameters was based on the previously reported average PCa diameter of 10 mm [16], and the results of our manual segmentation in the aforementioned 524 lesions in which the average maximum PCa diameter proved to be 23 mm.

### Manual segmentation

An expert uroradiologist (D.Y., 8 years of experience) manually segmented all 524 lesions on the axial T2-weighted sequence and on the spatially matched axial ADC map according to PI-RADS version 2.1 criteria [17]. Using ITK-SNAP [18], each lesion was segmented on a slice-by-slice basis. The total segmentation process was timed to later provide an average time per segmented lesion. The radiologist was blinded to all clinical information including pathological results. Figure 3B shows an example of a manual segmentation.

### Radiomics features

Radiomics features were extracted using total VOI–based calculation of the DLM auto-fixed VOIs (with 13 different diameters of the initial spherical VOI) and the manually segmented VOIs. All sequences included in the bpMRI studies (T2-weighted, DWI, and ADC) were used for this extraction which resulted in a total of 14 radiomics datasets based on the same set of 524 lesions. Each radiomics dataset consisted of 644 different features (belonging to 6 different feature groups, 8 for the manual model) calculated with PyRadiomics v3.0.1 [19]. All features that were extracted belong to the standard PyRadiomics set, and their names and descriptions can be found in the documentation (or website) [19]. The feature groups consisted of first-order or statistic-based features that try to distinguish voxel intensity distribution and five texture-based feature groups: gray-level co-occurrence matrix (GLCM), gray-level run-length matrix (GLRLM), gray-level size-zone matrix (GLSZM), gray-level dependence matrix (GLDM), and the neighboring gray-tone difference matrix (NGTDM) features. Additionally, the manual segmentation–based model included the features from the shape-based 2D and shape-based 3D PyRadiomics groups. Distance to neighbor specification for the texture matrices was kept to one, and no weighting was applied meaning the averages of separate matrices were returned. Features were calculated in a forced 2D extraction since no resampling was applied to the multi-center dataset and 3D calculation is expected to lead to less robust features due to anisotropic voxels and large differences in the slice dimension (*z*-axis) [20]. The use of forced 2D extraction and a fixed distance to neighbor of one resulted in a texture feature (GLCM, GLSZM, GLRLM, NGTDM, GLDM) connectivity of eight. Image-based normalization was applied and gray levels were discretized in fixed bin levels (bin width 30, resulting in the number of gray-level bins ranging from 30 to 130) [20]. No PyRadiomics image filters were applied due to previously proven insufficient impact [12].

### Bayesian model optimization

Radiomics feature selection was done using multivariate joint mutual information maximization [21], and relevant features were fed to an extreme gradient boosting model [22]. Before optimization and training of the total feature selection and model pipeline, the datasets were split into training and test datasets. A 4:1 training to test ratio was used for each of the 14 datasets (13 DLM, 1 manual). The training dataset consisted of 419 lesions (155 CS PCa, 264 non-CS), and the test dataset comprised 105 lesions (49 CS PCa, 56 non-CS). The test dataset was only used once at the end and kept completely separate from the training dataset. Feature selection and hyperparameter optimization were performed using Bayesian optimization which has shown excellent reproducibility and to be less susceptible to biased results [23]. Using Optuna [24], an automated hyperparameter optimization framework, Bayesian optimization was implemented as a sequential model-based optimization. The Optuna objective function consisted of a nested randomized five-times-repeated k-fold split for feature selection followed by a 10-fold cross validation of the model hyperparameters and the optimal number of trees. By optimizing the average area under the precision-recall curve which resulted from the 10-fold model cross validation, Optuna can iterate to achieve the best set of features and parameters. Use of the randomized nested split for joint mutual information maximization ensured a robust feature selection. The best-performing sets of parameters (features selected, model hyperparameters) for each of the 14 training datasets were then tested on the test dataset.

### Statistical analysis

The diagnostic performance metrics of each of the 14 datasets were evaluated in the test datasets. Multi-reader, multi-case analysis (iMRMC, version 4.0.1[25]) was performed on the DLM auto-fixed-based models and the expert manual segmentation–based model. When estimating *p* value, variances, and confidence intervals, iMRMC accounts for variability and correlations between models and test cases. Areas under the receiver operating characteristic curve (AUCs) were calculated for all of the test results, and 95% confidence intervals (CI) were estimated. Finally, the best-performing DLM auto-fixed–based models were compared to the expert manual segmentation–based model.

## Results

### 3D U-Net development

The total training of the 3D U-Net took 3 h. Weighted categorical cross-entropy was used as a loss function with an Adam optimizer, a weight of 0.5 to the background class, and a weight of 1 for the target class. The trained model reached a Dice score of 0.9683 on a randomly selected, held-out validation set of a random set of 10% (train: 105/valid: 11) of the data.

### Expert manual segmentation

The average time spent on a single manual lesion segmentation was approximately 5 min for the total process (i.e., segmenting each slice where the lesion occurs). The radiomics model based on the expert manual segmentation (booster: gbtree, boosting rounds: 9, max depth: 3, features: 31) achieved a test AUC of 0.62 (95% CI: 0.52–0.73). Test sensitivity and specificity of the expert manual segmentation was 0.84 (95% CI: 0.73–0.94) and 0.45 (95% CI: 0.32–0.57) at an optimized threshold (0.181). The final model feature list can be found in Electronic supplementary material 3.

### DLM auto-fixed VOI exploration

**Fig. 5:**
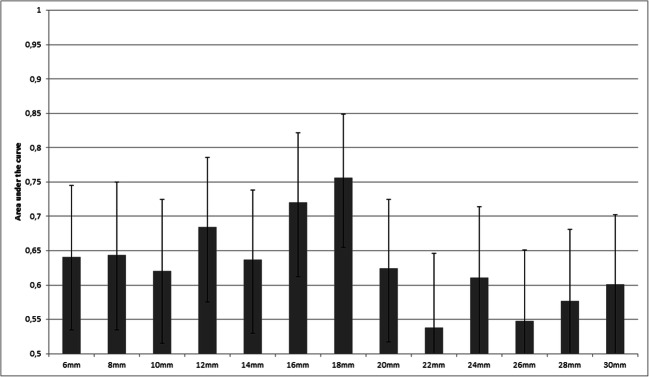
Deep learning masked auto-fixed VOI to extract bpMRI radiomics features for CS PCa: comparison of the performance of 13 different initial diameters of the auto-fixed VOI in the test set, expressed with AUCs and 95% confidence intervals (error bars)

**Fig. 6:**
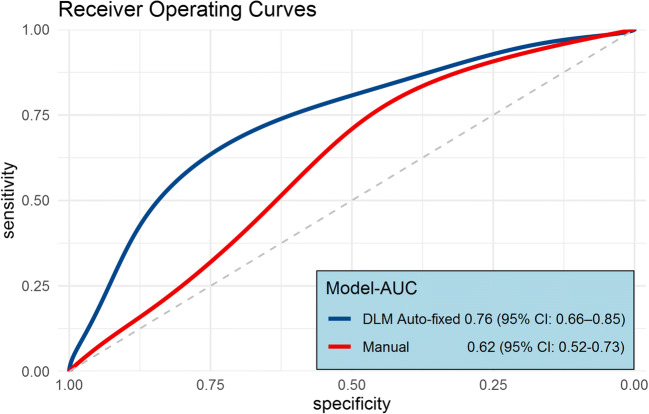
Test set smoothed ROC curves for the optimal DLM auto-fixed VOI model (initial 18–mm VOI diameter) and the model based on the expert manually segmented VOI as input

Figure 5 shows the diagnostic performance of DLM auto-fixed VOI bpMRI models for CS PCa, according to 13 different diameters of the initial auto-fixed spherical VOI. The highest scoring model (18-mm diameter, booster: gbtree, boosting rounds: 17, max depth: 4, features: 22) achieved an AUC of 0.76 (95% CI: 0.66–0.85) which was significantly higher (*p* = 0.0198) than that of the manual segmentation model’s AUC of 0.62 (95% CI: 0.52–0.73). The second best scoring model (16-mm diameter) achieved an AUC of 0.72 (95% CI: 0.62–0.81) which was not significantly different (*p* = 0.051) from the AUC of the manual segmentation model. Corresponding test sensitivity and specificity for the best model were 0.76 (95% CI: 0.63–0.86) and 0.71 (95% CI: 0.59–0.84) at an optimized threshold (0.385). Figure 6 shows the ROC curves of the best DLM model and the expert manual segmentation–based model. The model feature list for the 18-mm model can be found in Electronic supplementary material 4.

## Discussion

This study showed that our DLM auto-fixed VOI method is substantially faster than a manual-based method and is more accurate at diagnosing CS PCa. An initial diameter of 18 mm for the DLM auto-fixed VOI provided the highest diagnostic performance. Since the newly developed DLM auto-fixed VOI method only requires a single mouse click on the location that visually has the lowest ADC (which can typically be done within 10 s) and a complete manual segmentation is considerably more laborious (which typically takes approximately 5 min, > 97% increase compared to auto-fixed), the former is far more attractive to be used in clinical workflows and studies with larger datasets.

It can be argued that the DLM auto-fixed VOI model outperformed the complete manual VOI segmentation model, because the best-performing initial auto-fixed VOI diameter (18 mm) could be selected among no less than a total of 13 different diameters in the optimization experiment. Therefore, it can be asserted that this 18-mm diameter only applies to the current dataset without generalizability to others. However, because of the use of a large and diverse multi-center dataset (MRI data from 9 different medical centers, acquired with 8 different MRI systems [both 1.5 T and 3.0 T] from 2 different vendors, within a time span of 7 years), this argument seems not valid. Furthermore, only published standardized radiomics post-processing (image-based normalization and gray-level discretization) was used, and no elaborate post-processing or transformations were performed. Interestingly, a previous study by Wolters et al [16] reported the average PCa lesion diameter to be 10 mm. However, since lesions are often not perfectly spherical and may be elongated in a certain direction (e.g., the lesion shown in Fig. 3), only VOI diameters larger than 10 mm are expected to contain the necessary, relevant lesion information. Based on theory and their formulas, texture features should be better at extracting spatial information from bigger volumes than a small group or single voxels. This hypothesis is confirmed by the fact that the 6-mm (smallest diameter) auto-fixed VOI model used only 2 texture features, while the 18-mm auto-fixed VOI model used 8 texture features out of a total of 11 features. However, when the spherical VOI diameter is increased too much, the non-relevant regular prostate tissue information starts to impact performance. This balance (i.e., more than 10 mm, but not too much) is shown in Fig. 4 where a performance peak exists around a VOI diameter of 18 mm, and which diminishes for lower and higher diameters. Finally, the prediction that lesion edge information and healthy tissue might be helpful for radiomics could further explain the difference between manual and DLM. Although hard to prove exactly, besides the significantly better score, the gradient and sharpness of lesion edges combined with healthy tissue appear to improve PCa radiomics.

Several previous studies attempted to develop an algorithm for the diagnosis of CS PCa on MRI using radiomics [4, 26]. However, all of these studies used a manual segmentation or an automatic segmentation originally based on manual segmentations as input in their radiomics models [4] which still requires the time-consuming and tedious task of initially manually segmenting the lesions. The DLM auto-fixed VOI method is not limited by the availability of manual segmentations and allows for a faster analysis. To the best of our knowledge, there are no other studies that applied a deep learning–based semi-automatic segmentation technique combined with radiomics modeling for the diagnosis of CS PCa on mpMRI. Nevertheless, some comparisons can be made with the study by Brunese et al [27] that aimed to predict PCa Gleason scores through a deep neural architecture exploiting a set of radiomics features. While still based on manual segmentations, Burnese et al [27] did combine a set of radiomics features with a deep learning approach to predict Gleason scores. Their approach and specifically the prediction model had a much larger deep learning component since our model only contained radiomics features. This might also be reflected in their accuracy which averaged around 0.97. However, a much more plausible explanation for this relatively high accuracy in Burnese et al’s study [27] is the low number of patients (*n* = 62), no hold-out test set, and the use of accuracy, which is susceptible to label imbalances [28], without reporting the initial label distribution. Another study with some overlap with the currently proposed technique was done by Jing-Wen et al [29]. They developed a semi-automatic segmentation approach based on deep learning which was then used to calculate radiomics features. Similar to the present study, their approach led to a major time reduction when compared to manual segmentation and also managed to score higher than the manual-based model. However, the study by Jing-Wen et al [29] involved dual-energy computed tomography in gastric cancer, which differs considerably with the clinical setting of the present study.

The present study had some limitations. First, 320 of the 524 included lesions were non-CS entities which led to a minor label imbalance. However, this was accounted for by introducing a weight factor to the Bayesian optimization with a parameter space around the sum of the non-CS entities divided by the sum of the CS PCa lesions in the training set. Additionally, the use of AUC under the precision-recall curve as optimization metric for the Optuna objective function solves the remaining label imbalance issues. Second, the current dataset consisted of lesions in all prostate zones. Since radiomics features are significantly different in each zone [30], future more complex radiomics models, which can use the new segmentation technique as proposed in this current work, need to be investigated to account for these differences. Additionally, the optimization of the DLM diameter was performed for the total dataset and not each separate zone. A small experiment with the test data split for each zone did not show any differences in model performance in each zone. However, performance could perhaps improve if correctly optimized. Nonetheless, the deep learning zonal segmentation required for this optimization is outside of the scope of the current work.

In conclusion, a DLM auto-fixed VOI segmentation can provide a potentially more accurate diagnosis of CS PCa than expert manual segmentation while also reducing expert time investment by more than 97%.

## Supplementary Information

ESM 1 (DOCX 35 kb)

## Abbreviations

ADC: Apparent diffusion coefficient
AUC: Area under the curve
bpMRI: Biparametric magnetic resonance imaging
CI: Confidence interval
CS: Clinically significant
DLM: Deep learning masked
ISUP: International Society of Urological Pathology
mpMRI: Multiparametric magnetic resonance imaging
PCa: Prostate cancer
PI-RADS: Prostate imaging-reporting and data system
PSA: Prostate-specific antigen
ROC: Receiver operating curve
TRUS: Transrectal ultrasound
VOI: Volume of interest

## Declarations

### Guarantor

The scientific guarantor of this publication is Derya Yaka, MD PhD.

### Statistics and Biometry

No complex statistical methods were necessary for this paper.

### Methodology

• retrospective

• experimental

• multi-center study
