# Supplementary material for: A deep learning masked segmentation alternative to manual segmentation in biparametric MRI prostate cancer radiomics
Source: Eur Radiol. 2022 Apr 14;32(9):6526–35. doi: 10.1007/s00330-022-08712-8 (PMC9381625; doi:10.1007/s00330-022-08712-8)
Supplement: Supplementary file 1 — (DOCX 35 kb) [file 330_2022_8712_MOESM1_ESM.docx]

**Electronic Supplementary Material**

**Electronic supplementary material 1. Patient Data**

All patients (n=930) in the multi-center dataset were scanned between 2014 and 2020 because of a clinical suspicion of PCa. MRI scans were acquired using either Siemens or Philips 1.5- or 3-T systems (ESM Table 1).

| **Name** | **Vendor** | **Type of MRI system** | **Patients** |
| --- | --- | --- | --- |
| Hospital A | Philips | Ingenia 3T, Achieva 1.5T, Intera 1.5T | 572 |
| Hospital B | Siemens | Skyra 3T, Prisma 3T, Aera 1.5T | 164 |
| Hospital C | Siemens | Avanto 1.5T | 26 |
| Hospital D | Philips | Achieva 1.5T | 96 |
| Hospital E | Siemens | Avanto 1.5T | 15 |
| Hospital F | Siemens | Aera 1.5T | 19 |
| Hospital G | Philips | Ingenia 3T, Achieva 1.5T | 12 |
| Hospital H | Siemens | Espree 1.5T | 19 |
| Hospital I | Siemens | Espree 1.5T | 7 |

**ESM Table 1: Multi-center dataset location overview and patient distribution**

Overview of the nine different hospitals with the different MRI systems (vendor, type) and number of patients that were potentially eligible for inclusion. Hospitals A and B are the two tertiary care academic institutions.

The following sequences were acquired: T2-weighted (in-plane resolution range: 0.23 – 0.78 mm, slice thickness: 3-5 mm, axial, sagittal, and coronal acquisitions), diffusion-weighted imaging (in-plane resolution range: 0.85 – 2.19 mm, slice thickness: 3-5 mm, axial acquisition, b-values: 50, 400, 800, 1400 s/mm^2^ and mono-exponentially calculated ADC). A total of 1151 lesions were assigned prostate imaging - reporting and data system (PI-RADS) v2.0 scores[1] by two experienced uroradiologists (*BLINDED* 8 years’ experience, *BLINDED* 10 years’ experience) in clinical routine. Around half of the 1151 lesions was graded independently by one radiologist (blinded for pathological results and clinical follow-up) with the rest graded by the other radiologist(also blinded), no consensus decisions were made. Final distribution: PI-RADS 0/1/2: n=310, PI-RADS 3: n=170, PI-RADS 4: n=441, PI-RADS 5: n=230. Lesions were located in all zones of the prostate: peripheral zone: n=595, transition zone: n=272, central zone: n=7, anterior fibromuscular stroma: n=12, other (combination of zones): 265. Biopsies were performed by either targeted MRI-transrectal ultrasound (TRUS) fusion biopsy, cognitive fusion biopsy, in-bore MRI targeted or non-targeted TRUS biopsy. While prostatectomy specimens were also used for pathological correlation of the lesions detected on MRI. Using the obtained tissue specimens, dedicated genitourinary pathologists from the 2 tertiary care academic institutions assigned international society of urological pathology (ISUP) grades[2].

|  | **T2-Weighted - Sagittal** | | | **T2-Weighted - Coronal** | | |
| --- | --- | --- | --- | --- | --- | --- |
|  | *Min* | *Max* | *Median* | *Min* | *Max* | *Median* |
| Repetition Time | 3500 | 8350 | 4421 | 2930 | 10070 | 5200 |
| Echo Time | 95 | 120 | 120 | 80 | 123 | 80 |
| Field of View | 10 | 20 | 16 | 8 | 20 | 13 |
| Number of Excitations | 1 | 3 | 1 | 1 | 4 | 1 |
| Interslice gap | 3 | 4 | 3.3 | 3 | 3.5 | 3 |
| Acquisition Matrix rows | 256 | 404 | 400 | 256 | 552 | 440 |
| Acquisition Matrix columns | 205 | 372 | 313 | 205 | 445 | 440 |
|  | **T2-Weighted - Transverse** | | | **Diffusion-weighted imaging** | | |
|  | *Min* | *Max* | *Median* | *Min* | *Max* | *Median* |
| Repetition Time | 2744 | 12830 | 4515 | 1726 | 10200 | 5817 |
| Echo Time | 95 | 120 | 120 | 55 | 130 | 99 |
| Field of View | 9 | 30 | 26 | 7.5 | 32 | 16 |
| Number of Excitations | 1 | 6 | 1 | 1 | 11 | 3 |
| Interslice gap | 2.2 | 4 | 3 | 3 | 4.2 | 3 |
| Acquisition Matrix rows | 224 | 878 | 768 | 80 | 192 | 116 |
| Acquisition Matrix columns | 205 | 605 | 585 | 44 | 249 | 116 |

**ESM Table 2: MRI setting ranges for separate sequences**

Detailed overview of a number of MRI settings and their ranges for the multi-center dataset using minimum, maximum and the median.

**Electronic supplementary material 2. Study Population**

The PI-RADS distribution of the study population was: PI-RADS 0/1/2: n=82, PI-RADS 3: n=104, PI-RADS 4: n=252, PI-RADS 5: n=86. The age of these 427 patients ranged from 62 years to 88 years with a median of 69 years. Prostate specific antigen (PSA) levels ranged from 0.73 µg/L to 112 µg/L with a median of 8.55 µg/L, while PSA density levels ranged from 0.019 to 2.04 µg/L^2^ with a median of 0.188 µg/L^2^. Average time between MRI and biopsy for these 427 patients ranged from 0 to 172 days with a median of 30 days. Each of the 524 lesions underwent targeted biopsy. This also applied to each single lesion in patients with multiple lesions. ISUP grades were based on MRI-TRUS fusion biopsy in 456 lesions, on cognitive fusion biopsy in 45 lesions, and on in-bore MRI targeted biopsy in 23 lesions. Number of biopsy cores ranged from 1 to 7 with a median of 3 cores. The final study population contained 318 non-significant lesions and 206 significant lesions, the ISUP grade distribution was: ISUP 1: n=318, ISUP 2: n=127, ISUP 3: n=46, ISUP 4: n=19, ISUP 5: n=14. Of these biopsies 216 could be defined as MRI first, 104 had a history of negative TRUS biopsies, 107 an increasing PSA in general or during active surveillance and the remaining 97 biopsies were either for staging purposes or a coincidence.

**Electronic supplementary material 3. Feature list manual segmentation based radiomics model**

| **Feature** | **Sequence** | **Details** |
| --- | --- | --- |
| original_glrlm_HighGrayLevelRunEmphasis | DWI | b-value : 1400 s/mm² |
| original_glszm_HighGrayLevelZoneEmphasis | DWI | b-value : 1400 s/mm² |
| original_glrlm_ShortRunHighGrayLevelEmphasis | DWI | b-value : 1400 s/mm² |
| original_gldm_HighGrayLevelEmphasis | DWI | b-value : 1400 s/mm² |
| original_glcm_Autocorrelation | DWI | b-value : 1400 s/mm² |
| original_firstorder_Mean | ADC |  |
| original_firstorder_10Percentile | ADC |  |
| original_firstorder_Median | ADC |  |
| original_firstorder_Minimum | ADC |  |
| original_firstorder_RootMeanSquared | ADC |  |
| original_firstorder_90Percentile | ADC |  |
| original_glrlm_RunEntropy | DWI | b-value : 1400 s/mm² |
| original_firstorder_TotalEnergy | DWI | b-value : 1400 s/mm² |
| original_glcm_ClusterProminence | T2 | Sagittal plane |
| original_gldm_SmallDependenceHighGrayLevelEmphasis | DWI | b-value : 1400 s/mm² |
| original_glszm_SmallAreaHighGrayLevelEmphasis | DWI | b-value : 1400 s/mm² |
| original_glcm_ClusterProminence | DWI | b-value : 1400 s/mm² |
| original_firstorder_Entropy | DWI | b-value : 1400 s/mm² |
| original_glrlm_LongRunHighGrayLevelEmphasis | DWI | b-value : 1400 s/mm² |
| original_glcm_SumEntropy | DWI | b-value : 1400 s/mm² |
| original_glcm_DifferenceAverage | DWI | b-value : 1400 s/mm² |
| original_glcm_SumSquares | DWI | b-value : 1400 s/mm² |
| original_glcm_JointAverage | DWI | b-value : 1400 s/mm² |
| original_glcm_DifferenceEntropy | DWI | b-value : 1400 s/mm² |
| original_glcm_SumAverage | DWI | b-value : 1400 s/mm² |
| original_shape_SurfaceArea | T2 | Coronal plane |
| original_glcm_ClusterShade | T2 | Coronal plane |
| original_firstorder_MeanAbsoluteDeviation | DWI | b-value : 1400 s/mm² |
| original_firstorder_InterquartileRange | DWI | b-value : 1400 s/mm² |
| original_glcm_Id | DWI | b-value : 1400 s/mm² |
| original_shape_SurfaceArea | T2 | Transverse plane |

**Electronic supplementary material 4. Feature list 18MM DLM radiomics model**

| **Feature** | **Sequence** | **Details** |
| --- | --- | --- |
| original_glszm_LargeAreaHighGrayLevelEmphasis | T2 | Transverse plane |
| original_firstorder_10Percentile | ADC |  |
| original_glszm_HighGrayLevelZoneEmphasis | DWI | b-value : 400 s/mm² |
| original_firstorder_Median | ADC |  |
| original_firstorder_Energy | T2 | Transverse plane |
| original_firstorder_TotalEnergy | DWI | b-value : 400 s/mm² |
| original_glrlm_ShortRunHighGrayLevelEmphasis | DWI | b-value : 400 s/mm² |
| original_firstorder_Energy | ADC |  |
| original_glszm_HighGrayLevelZoneEmphasis | T2 | Sagittal plane |
| original_firstorder_Mean | ADC |  |
| original_glcm_DifferenceEntropy | DWI | b-value : 50 s/mm² |
| original_glszm_SmallAreaHighGrayLevelEmphasis | DWI | b-value : 1400 s/mm² |
| original_glszm_SmallAreaHighGrayLevelEmphasis | DWI | b-value : 400 s/mm² |
| original_glcm_SumEntropy | DWI | b-value : 1400 s/mm² |
| original_firstorder_TotalEnergy | T2 | Transverse plane |
| original_glcm_JointAverage | DWI | b-value : 400 s/mm² |
| original_firstorder_RootMeanSquared | ADC |  |
| original_firstorder_90Percentile | T2 | Transverse plane |
| original_firstorder_90Percentile | ADC |  |
| original_glcm_Id | DWI | b-value : 1400 s/mm² |
| original_gldm_GrayLevelVariance | DWI | b-value : 1400 s/mm² |
| original_glcm_Idm | DWI | b-value : 1400 s/mm² |

**References**

1. Weinreb JC, Barentsz JO, Choyke PL, et al (2016) PI-RADS Prostate Imaging - Reporting and Data System: 2015, Version 2. Eur Urol 69:16–40

2. Epstein JI, Egevad L, Amin MB, Delahunt B, Srigley JR, Humphrey PA (2016) The 2014 international society of urological pathology (ISUP) consensus conference on gleason grading of prostatic carcinoma definition of grading patterns and proposal for a new grading system. Am J Surg Pathol 40:244–252
